# Fracture of the Shaft of Femur in a Child

**Published:** 1878-02

**Authors:** S. J. Radcliffe

**Affiliations:** 1211 F St., N. W., Washington, D. C.


### Correspondence.

1211 F St., N. W.,<br>

Washington, D. C., Jan. 10th, 1878.

Prof. Frank H. Hamilton, M. D.

Dear Sir:—On reading your lecture on "Fracture of the shaft of the femur in children" in the "Medical Record" for Jan. 5, 1878, I was reminded of a case of nearly transverse fracture of the upper third of the thigh which occurred a short time ago in a child five years old and which I treated substantially as you describe, making reference to your "Treatise on Surgery."

It occurred far out of the way of assistance, I had no anæsthetic at hand, the piercing cries of the child were exceedingly distressing and I had great difficulty in adjusting the fracture and applying temporary apparatus. I went home, made the entire outfit myself —the long splints from light pine strips and the short splints from bark splints cut down to the proper size. I applied the dressing satisfactorily, with a three pound bag of sand as extension power. I examined the parts daily and saw that all the apparatus was kept in position. The child laid perfectly quiet and comfortably and scarcely whimpered during the time of treatment. In five weeks I took off the splints and bandages, examined and measured the limb in comparison with the other and I could discover no difference. The limb was perfectly straight and if there was any shortening it was not apparent to the eye or touch. I set him on his feet in six weeks, and after using a single crutch for two weeks more I discharged him and let him go on his own responsibility, and I am sure no one could tell, except from a slight elevation of bone substance around the fracture from hardened callous, that he ever had a fracture.

Yours Truly, S. J. Radcliffe, M. D.

---

New York, 43 W. 32.
Jan. 14, 1878.

*Editor Buffalo Medical and Surgical Journal:*

My Dear Doctor:—The plan of treating fractures of the thigh in children described in the lecture referred to by Dr. Radcliffie, is so simple and efficient, and has, under my observation, saved the little patients from so much suffering, that I feel it my duty to send you this note, with permission to publish it if you see fit. I am sure Dr. Radcliffe would not object to its publication.

With sentiments of esteem, I remain yours truly,

Frank H. Hamilton.

———:o:———

The first number of the *Michigan Medical News*, a Semi-Monthly Journal, is before us. From the number and character of the men who appear as editors we are led to expect a success, and herewith present our congratulations.

